# Users’ satisfaction with the primary health care information system in Croatia: a cross-sectional study

**DOI:** 10.3325/cmj.2012.53.60

**Published:** 2012-02

**Authors:** Lidija Andrijašević, Petra Angebrandt, Josipa Kern

**Affiliations:** Department of Medical Informatics, University of Zagreb, School of Medicine, Zagreb, Croatia

## Abstract

**Aim:**

To evaluate the primary health care information system from the general practitioner's (GP) point of view.

**Methods:**

Sixty-seven Croatian GPs were distributed a questionnaire about characteristics of the GP’s office, overall impression of the application, handling of daily routine information, more sophisticated information needs, and data security, and rated their satisfaction with each component from 1 to 5. We also compared two most frequently used applications – application with distantly installed software (DIS) and that with locally installed software (LIS, personal computer-based application).

**Results:**

GPs were most satisfied with the daily procedures and the reminder component of the health information system (rating 4.1). The overall impression ranked second (3.5) and flexibility of applications followed closely (3.4). The most questionable aspect of applications was data security (3.0). LIS system received better overall rate than DIS (4.2 vs 3.2).

**Conclusion:**

Applications received better ratings for daily routine use than for overall impression and ability to get specific information according the GPs’ needs. Poor ratings on the capability of the application, complaints about unreliable links, and doubts about data security point to a need for more user-friendly interfaces, more information on the capability of the application, and a valid certificate of assessment for every application.

The application of information and communication technology (ICT) to health care has changed the current medical practice. The most prominent aspect of ICT is the electronic health record (EHR). Some authors confirmed that the EHR indeed led to higher performance ratings on certain quality measures (1,2), whereas others were suspicious about it (3,4). The EHR systems offer better management of clinical data and improvement of management and prevention of chronic diseases (5). Both physicians and patients generally have a positive attitude toward the EHR (6). However, both are concerned about issues like privacy, physician-patient relationship, cost, time, and training needs. Only 10.2% of physicians in ambulatory care declared interest in using information technology in their daily practice (7).

Further potential applications of an ICT-based information system in general practice are electronic reminders and decision support. Several studies show positive effects of electronic reminders: a recall system can result in higher immunization rates against seasonal influenza of high-risk groups (8), computerized body mass index charts increase the likelihood that physicians would diagnose obesity and refer patients to treatment (9), and decision support in electronic prescribing leads to more responsible prescribing (10-13). However, the use of electronic reminders does not seem to improve the quality of care in diabetes and coronary artery disease (14).

Implementation of ICT leads to decreased financial expenses (15,10). Negative implications of modern technology include increased duration of consultation, more stress for the physicians (16), and increased data entry at least at the beginning of ICT use (17). Computers in the examination room could affect the patient-centered practice, shorten the patient-physician interaction and interfere with it, particularly in the psychosocial and emotional aspects. Looking at the screen is particularly disruptive and often leads to poor eye contact with the patient (18). Still, the most recent studies have not found any negative influence of ICT on the physician-patient relationship, even with psychiatric patients (19,20). Finally, in spite of different attitudes toward an ICT-based health information system in clinical practice, EHR serves as a cohesive clinical basis and allows physicians to carry out research or analyze their professional activities more easily (1).

The general challenge for developers of ICT applications in health care is to make them suitable for health professionals’ information needs. Users’ satisfaction or dissatisfaction with ICT applications is one of the most important issues to be considered. There is a number of ICT applications in health care worldwide, and Croatia is not an exception. Primary Health Care Information System (PHCIS) was one of the first e-Government activities in Croatia. It started in 2002 and was fully implemented in 2008. Designed to cover the primary health-care information needs, the PHCIS consists of the central EHR repository (the so-called first level), accessible by locally installed applications in GP’s offices, and the second level for authorized users only (21-23). Development of the PHCIS was initiated by the Ministry of Health and Social Welfare and the Croatian Health Insurance Institute. The tender was announced early in 2003 and its winner, an ICT enterprise, included public health experts and several GPs to serve as health professional consultants in the development. This group primarily worked on the core system or the first level. The second level involved a number of smaller ICT companies working on the local information needs, ie, information needs of GPs in their daily work with the patients. The users of second level ICT applications were obliged to communicate with the first-level users – to send and receive data. There were eight available ICT applications (status on October 12, 2011) enabling the end-users (GPs and nurses) to enter patients’ data and use it in their daily work, as well as to create reports for administrative, professional, and other purposes. Any GP’s office could choose one of the certified ICT applications from the list on the PHCIS web site (*http://www.cezih.hr*). Two basic approaches in the development of the second level ICT applications were distantly installed software (DIS) and locally installed software (LIS). DIS was web-based approach installed on distant servers connected to the first level of PHCIS, outside of the GP’s office, but the GP could access it by standard browser through the virtual private network. LIS was installed on computers in the GP’s office connected directly to the first level of PHCIS. Both applications were able to send some selected patients’ data to the first level of PHCIS.

The aim of this study was to analyze the second (local) level of PHCIS from the users (GPs’) point of view and the specific aims included the following: 1) to find out specific functions of ICT applications that were thought to be appropriate or problematic and 2) to compare two conceptually different approaches to the development of the local applications.

## Methods

### Settings

We selected a purposeful sample of 67 GP’s offices in four cities in Croatia (Zagreb, Karlovac, Đakovo, and Ivanić Grad). The GP office (with a physician and a nurse) was chosen as the study unit. The questionnaire was given to the physician. The questions were related to the PHCIS used in GP’s offices in Croatia.

The response rate was 64% (43 offices). Three ICT applications were reported but one of them only once, so the GP using it was excluded from further analysis. One of these applications was based on web technology (software installed on distant server and usable to GP’s office known as distantly installed software – DIS), and the other based on Windows technology installed locally on the personal computer in the GP’s office (locally installed software – LIS). It should be noted that GPs are not laymen in ICT and medical informatics – medical curriculum at the School of Medicine (University of Zagreb, as well as other Croatian universities) includes medical informatics topics based on the Recommendations of International Medical Informatics Association on Education in Biomedical and Health Informatics (knowledge and skills for information technology users) (24).

### Data collection and analysis

The questionnaire included five groups of questions that were used to gather the following data: 1) the number of patients registered in the GP’s office, number of patients seen daily, number of years that the GP worked in the office, and the extent of the ICT application use (questions 1-5); 2) the GP’s general impression of the software regarding issues such as simplicity of data input, clarity of data output, and appropriate display of patient history or other parts of the medical record (question 6); 3) appropriateness of the ICT application for administrative procedures, reminders, and decision support relevant for daily work, like referrals, prescriptions, predefined reports, and drug information (questions 7-12); 4) the flexibility of the application in the sense of its potential to produce more sophisticated *ad hoc* data reports or data analysis, or to export data useful for quality assurance (question 13); 5) GP’s impression of appropriateness of data protection (question 14). For each question, GPs were asked to select a number that reflected his/her perceived agreement with the statement on a rating scale from 1 (strongly disagree) to 5 (strongly agree). They were also allowed to add any comment or suggestion (problems, drawbacks, or satisfaction) (web-extra material)(web extra material 1).

Data analysis included creating contingency tables and calculating medians and range for data describing each GP’s office, and averages for rates. Differences were tested by applying standard statistical tests (Fisher exact test, Kruskal-Wallis test). We used SAS software (SAS Institute Inc., Cary, NC, USA) and *P*-value was considered significant at 0.05.

## Results

### Description of GP’s offices

The first group of questions was analyzed in order to show general characteristics of GP’s office. GP’s offices were divided into two groups according to the type of applications they used (DIS and LIS). There were no significant differences between them in the number of years of GP’s work experience (χ^2^ = 1.525, *P* = 0.217), number of patients (χ^2^ = 0.699, *P* = 0.403), and number of patients (χ^2^ = 0.014, *P* = 0.906) (Table 1). The extent of the use of different ICT applications (Table 2) was nearly the same for each group (Fisher exact test, *P* = 0.612). Interestingly, 8 (19%) GPs used electronic and paper-based medical record in parallel.

**Table 1:** General characteristics of general practitioners (GP) included in the study (median and range)

Information and communication technology	Years of GP’s work experience in the office	*P*	Number of patients registered	*P*	Number of patients per day	*P*
Distantly installed software	28 (4-38)	0.217	1730 (1300-2600)	0.403	55 (32-80)	0.906
Locally installed software	20 (3-39)		1900 (1300-2400)		50 (30-75)	
Total	23 (3-39)		1855 (1300-2600)		50 (30-80)	

**Table 2:** Extent of use of electronic health record (EHR) among general practitioners (GP)

	No. (%) of physicians who use		
Information and communication technology application	EHR only*	EHR partially^†^	EHR and paper-based health record in parallel^‡^
Distantly installed software	9 (43)	8 (38)	4 (19)
Locally installed software	11 (52)	6 (29)	4 (19)
Total	20 (48)	14 (33)	8 (19)

*GPs who circled the answer a) completely – “I do not use the paper form at all” (Question 5).

†GPs who circled the answer b) partially – some of data are recorded in electronic, some in paper records (Question 5).

‡GPs who circled the answer c) double-data records – the same data are recorded in both electronic and paper records (Question 5).

### General rating and comparing of applications

The highest rating in each approach was given to routine procedures (4.1), followed by overall impression (3.5) and flexibility of applications (3.4). The most problematic part of applications was data security (3.0) (Table 3). Taking into account all the rates for all the questions (total average rate), DIS application achieved a lower total average rate (3.2) than LIS (4.2). There were 20 (48%) participants using DIS application and 14 (33%) using LIS application who expressed a complete lack of confidence in data security (rates 1 and 2). However, 10 (24%) participants using DIS and 18 (43%) participants using LIS application considered the data completely safe (rates 4 and 5).

**Table 3:** Rating of specific aspects of the information and communication technology applications (average rate)

Information and communication technology application	Overall impression	Routine procedures	More sophisticated information needs	Data security
Distantly installed software (DIS)	3.1	3.6	2.9	2.8
Locally installed software (LIS)	4.0	4.5	4.0	3.2
*P*-value (difference between DIS and LIS), Kruskal-Wallis test	0.007	0.001	0.025	0.532
Total	3.5	4.1	3.4	3.0

### Qualitative comparisons – suggestions, comments, opinions

We received more comments on DIS than on LIS application (8 and 5 comments, respectively). Comments on LIS were related to the problem of communication with the core of the system, security issues, and a proposal for upgrading the system with new possibilities like e-prescription, e-discharge letter, etc. Comments on DIS were mostly communication-related, like broken links with the core of PHCIS and slow communication (5 comments), too much clicking to get information (1 comment), or security of data (2 comments). Prescribing and information on drugs (reminder on doses, indications, interactions, and similar) were classified as good (1 comment). Several GPs pointed to insufficient information for users of particular ICT application (not clear manual, not clear interface, not enough information how to use the application).

## Discussion

The Croatian GPs gave their ICT applications good ratings for routine (administrative) information procedures (prescriptions, drug information, etc) and less good for overall impression and flexibility in getting information. The greatest concern reported was data security. Additionally, GPs reported problems with communication between the local and central parts of PHCIS, as well as having insufficient education to fully incorporate PHCIS in their daily work. In spite of a number of articles on the Croatian primary health care information system published in internationally available journals (1,21-23,25-32) and in several other sources (proceedings and reports), this study is the first evaluation of this system from the end-user point of view. Most previous studies pay attention to particular aspects of the health information system, like prevention and management of chronic diseases (5,33), financial expenses (15,34), access time (17), patient point of view (6,35), effect of electronic reminders (36-39), and quality of care, duration of consultation, stress for physicians, and overall problem of using modern technology (16,18). Having in mind that any information system is only as good as the end-users rate it, we decided to start an evaluation based on user satisfaction.

### What is satisfactory and what are the challenges?

Our results showed that the skeleton of the information system could be considered satisfactory (daily routine medical procedures are covered by the system). However, flexibility of getting information and communication between GPs and information system in order to answer an *ad hoc* question, like “Why do I prescribe so many antibiotics?”, was recognized as insufficient. Moreover, GPs were not convinced in the security of patients’ data.

As we found the two applications mostly used in our sample, we decided to investigate if there were some differences in the GPs’ perception of their usefulness. There are conceptual differences between DIS and LIS, which may have influenced GPs’ rating of these two applications. When these differences are taken into account, together with the rating for each component of the application – this could serve as useful guide for companies that develop such applications. GPs’ rating of DIS application might have been influenced by their inability to use the application each time there is a problem with the internet connection. As seen from their comments, friendly interface and simple operability is crucial for the perception of application’s usefulness. Any issues dealing with security and/or reliable communication links could be solved only by certification of ICT products.

Limitations of the study were a relatively small sample size and use of only two applications out of the eight existing at the time of the publication. Deployment of an ICT-based PHCIS leads to changes in GPs’ daily work and demands more ICT knowledge and skills. Users should be educated about changes to the system and ways to manage such changes. Finally, it is desirable that educated GPs are involved in PHCIS development from the very beginning.
