# Supplementary material for: Users’ satisfaction with the primary health care information system in Croatia: a cross-sectional study
Source: Croat Med J. 2012 Feb;53(1):60–5. doi: 10.3325/cmj.2012.53.60 (PMC3284175; doi:10.3325/cmj.2012.53.60)
Supplement: Supplementary material [file CroatMedJ_53_s012.pdf]

Appendix.

Questionnaire

Dear Doctor,

As the experienced GP and user of ICT in your office, you are kindly asked for assistance in evaluation of the ICT application you use.

In front of you is a short questionnaire. Your answer to any particular question will be appreciated. The statement in questions 6-14, please, rate by 1 (strongly disagree), 2, 3, 4 or 5 (strongly agree). Comment or explanation of your rate is possible.

Any suggestion for improvement of the most problematic parts of the ICT application you use will be very much appreciated.

This questionnaire is completely anonymous.

1. ICT application you use:___________________________________
2. Number of years you have worked in the GP office:_____________
3. Number of patients registered in your office:__________________
4. Number of patient daily:___________________
5. Extent of usage of the ICT application (please circle):

   a) completely - I do not use a paper form at all

   b) partially – some of data are recorded in electronic, some in paper records

   c) double-data records - the same data are recorded in both electronic and paper record

Please rate and comment specific parts of the ICT application:

6. Design of application and screen appearance are user friendly – clearness and convenience of information you need

   1 2 3 4 5

   Comment: ____

7. General patient data (like allergies, marital status, occupation etc.) – useful and simple to input

1 2 3 4 5

Comment: ____

8. Patient history - simple data input, clear and useful the previously entered data

1 2 3 4 5

Comment: ____

9. Referrals - simplicity of writing referrals

1 2 3 4 5

Comment: ____

10. Prescriptions - simplicity of writing prescriptions, complete and up to date information on drugs

1 2 3 4 5

Comment: ____

11. Sick leaves, disability, excuse-notes, travelling expenses etc. - appropriate and easy to do

1 2 3 4 5

Comment: ____

12. Reports (daily, weekly, monthly) – appropriate and easy to do

1 2 3 4 5

Comment: ____

13. You can get information enabling you to supervise your work (e.g. to find information on the number of antibiotics prescriptions in case of respiratory infections in adults, or how many patients you have not meet for last three years, etc., or to make some other ad hoc created inquiry)

1 2 3 4 5

Comment: ____

14. Patient data are appropriately protected

1 2 3 4 5

Comment: ____

Your other suggestions, comment, opinions:

Web – extra (GPs` comments on LIS and DIS):

Program 1- DIS	Program 2- LIS
1. mistakes with ICD (International Classification of Diseases) codes, there is no sufficient and clear instructions from central health information system (CEZIH), too many mouse clicking what slows down the process.	9. very satisfied, only problems with transferring information and data base to central health information system (CEZIH)
2. the program is too slow. Data security is questionable.	10.There should be global network with out-patient department in local health centers and hospitals, laboratories, pharmacies. To be able to email discharge letter from a hospital center to a primary doctor .
3. the main screen appearance is not clear, dependence of PC, very few paper documents, too slow	11. data base is safe in PC, but users are not sure if it is safe in central health information system (CEZIH)
4. system is going off often, hard communication with central health information system (CEZIH)	12. more codes for sick-leave, good conditions for prescriptions, enough information about each drug and its usage
5. difficult communication with central health information system (CEZIH), the system is going off very often, too much clicking with mouse, switching to another application causes a problem with transfering patient data base.Data from invalid retirement are often lost or deleted without any cause. Very slow.	13. Data base are safe in PC, but users are not sure if it is safe in central health information system (CEZIH), users are wondering how many people have access to central health information system (CEZIH)????
6. there isn't enough instructions and information about usage of the ICT application and what are doctors options and possibilities, or the doctor has not got enough of it.	Program 3 (excluded from further analysis)<br>14. when the system is going off transferring of patient data is stopped and that stunned user work and makes efficiency lower, there is not enough education for doctors about application.

7. there is no enough information, for example, one user did not know that she/he can check out how many patients and which patient did not visit her/him in past 3 years.	15. better way for inputting data about patient allergies, more information about social patient's data, better clarity of data input, more possibilities for prescription of some magistral drugs, better control over the patient who were not in visit over 2 years, bigger insert of data about patient history and diseases
8. it should not be allowed one doctor to leave his own SMART card to another doctor in case of interchange in his out-patient department during vacation or sick- leave- that is the case in Local Health Centers. Program should be faster.	
